# Mortality of Alzheimer’s Disease and Other Dementias in China: Past and Future Decades

**DOI:** 10.3389/ijph.2023.1605129

**Published:** 2023-02-03

**Authors:** Bin Lv, Li Liang, Anan Chen, Hua Yang, Xiaolan Zhang, Fangfang Guo, Hairong Qian

**Affiliations:** ^1^ Department of Neurology, The First Medical Center, Chinese PLA General Hospital, Beijing, China; ^2^ Navy Clinical College, The Fifth School of Clinical Medicine, Anhui Medical University, Hefei, China; ^3^ Department of Outpatient, No.13 Cadre Santatorium of Beijing Garrison, Beijing, China; ^4^ The Second School of Clinical Medicine, Southern Medical University, Guangzhou, China

**Keywords:** dementia, mortality, epidemiology, aging, Alzheimer’s disease

## Abstract

**Objectives:** This study aimed to explore the distribution features and trends of dementia mortality in China from 2011 to 2020 and make a prediction for the next decade.

**Methods:** Mortality-relevant data were gathered from the Chinese Center for Disease Control and Prevention’s Disease Surveillance Points system. Joinpoint regression was applied to evaluate the trends.

**Results:** Crude Mortality Rate (CMR) of AD and other dementias increased from 3.7 per 100,000 to 6.2 per 100,000 in 2011–2020, with an Average Annual Percent Change (AAPC) of 5.3% (95% CI 4.4%–6.3%). Age-Standardized Mortality Rate (ASMR) slightly decreased from 5.0 per 100,000 to 4.1 per 100,000 in 2011–2020, with AAPC of −0.4% (95% CI −2.5%–1.8%). CMR will increase to 9.66 per 100,000 while ASMR will decline to 3.42 per 100,000 in the following decade.

**Conclusion:** The upward trend in CMR and downward trend in ASMR suggested the further development of population aging and dementia mortality in the past and future decades. In China, there were gender, urban-rural, regional and age differences.

## Introduction

Alzheimer’s disease (AD) and other dementias are acquired intellectual impairment disorders with cognitive dysfunction as the primary symptom. Over 55 million individuals worldwide have dementia, and there are nearly 10 million new cases every year [1]. By 2050, the number of dementia patients would increase to 152.8 million [2]. A national cross-sectional study showed that China had roughly 15.07 million people over 60 years old living with dementia, accounting for a quarter of all dementia patients worldwide [3]. AD and other dementia have become an increasingly serious public health and social problem.

Due to the disparity in society, economy, and demographic policy, different countries and regions have different abilities to diagnose AD, therefore the prevalence and mortality of dementia are also varied. A systematic review and meta-analysis published in 2020 showed that the prevalence of dementia was higher in Europe and North America than in South America, Asia, and Africa [4]. Because of the long-term improvements in education levels, lower population levels of vascular risk factors, and an overall decrease in stroke incidence [5–8], the prevalence of AD and other dementias declined in Europe and America [5, 9–12]. But in the world today, as the size of the population aged over 65 continues to increase, the mortality of AD and other dementias would grow. In America, official death certificates recorded 121,499 deaths from AD in 2019 [13]. According to the 2019 data from American Center for Disease Control and Prevention (www.cdc.gov), mortality for AD in America was 37 per 100,000, making AD the 6th-leading cause of death. Moreover, the deaths of dementia in America were estimated to increase by 16% during the COVID-19 pandemic [14]. In the UK, the Office for National Statistics (www.ons.gov.uk) reported that the AMSR for deaths due to dementia in England and Wales in 2019 was 115.1 per 100,000 people (66,424 deaths), which was significantly lower than the ASMR in 2018 (123.8 per 100,000 people, 69,478 deaths). However, COVID‐19 has already disproportionally impacted the population with dementia with an eightfold larger share of deaths [15, 16].

In Asia, the fastest growth in the elderly population is taking place. Japan was considered a super-aging country, the number of people with AD was expected to reach approximately 6.5–7 million by 2025 and 8.5 to 11.5 million by 2060 [17]. Japanese mortality of dementia increased from 15.3 per 100,000 people in 1999 to 69.0 per 100,000 people in 2016 [18]. South Korea had the fastest aging population in the world, as well as a low fertility rate. Both crude and standardized prevalence of dementia in Korea were increasing [19]. In 2003 and 2015, the crude prevalence of dementia per 100,000 Koreans aged ≥60 years was 135.63 and 2696.31, and the standardized rate were 126.41 and 2218.25. A report published by Statistics Korea (www.kostat.go.kr) showed the mortality of dementia in Korea increased 2.5 times between 2009 and 2019, pushing it from 13th to 7th as a cause of death.

In China, the overall age-adjusted and sex-adjusted prevalence was estimated to be 6.0% for dementia, 3.9% for AD, 1.6% for VaD, and 0.5% for other dementias among aged 60 years or older [3]. A previous study based on the Global Burden of Disease (GBD) estimated that Chinese mortality of AD and other dementias was 22.5 per 100,000 (95% CI 5.4–59.3) in 2019, and the mortality increased significantly between 1990 and 2019, pushing it from 10th to 5th place as a cause of death [20]. However, comprehensive mortality descriptions of AD and other dementias based on the Chinese national disease surveillance points system, especially stratified mortality differences, have rarely been performed in recent years. Based on data from the Chinese Center for Disease Control and Prevention’s Disease Surveillance Points system (CDC-DSP), this study aimed to identify the distribution characteristics of the mortality of AD and other dementia in China by age, gender, residence, and region, and to evaluate the trends in the mortality of AD and other dementias from 2011 to 2020. In addition, trends are predicted for the coming decade.

## Methods

### Data Sources

Mortality relevant data were gathered from the Chinese Center for Disease Control and Prevention’s Disease Surveillance Points system. This surveillance system was comprised of 605 points (208 urban points and 397 rural points) covering 323.8 million people, approximately a quarter of the total Chinese population All the surveillance sites perform mortality registration work in accordance with the unified standards and procedures described in the guidelines for surveillance in the DSP [21]. CDC has conducted several system evaluations and adjustments during the development of the DSP to ensure the reliability of the data. Underreporting adjustments are also made. The International Classification of Diseases 10 (ICD-10) [22] was used to code the data. Data were extracted by using the U087 identification code, which contained AD (G30), other dementias such as VaD (F01), non-specific dementia (F03), and not otherwise defined dementias (G31). Gender, age, place of residence (urban or rural), and region (East, Central and West China) were also extracted. Aging subgroups were divided into 5-year intervals from 60 to 84 years, and 85+ years.

### Statistical Analysis

Age-Standardized Mortality Rate (ASMR) was calculated using the population from the 2010 China Census provided by the National Bureau of Statistics of China (www.stats.gov.cn). Temporal trends in Crude Mortality Rate (CMR) and ASMR were examined by Joinpoint Regression models (version 4.9.0.0; National Cancer Institute). In 2000, Kim et al. [23] first proposed the Joinpoint regression model, which uses joinpoints to divide a long-term trend line into segments, and each segment is described by a continuous linear. This model uses the Z-test for the hypothesis of segmentation points. The principle is to first assume that there are no segmentation points, that is H0: there are 0 segmentation points, and then traditional linear regression can be used for analysis; H1: there is at least 1 segmentation point. If H0 is rejected, the test is then conducted to see if the difference between 1 segmentation point and n segmentation points is statistically significant, and so on. Annual Percent Change (APC) for each time segment and the Average Annual Percent Change (AAPC) for the whole study period, along with their 95% Confidence Intervals (CIs), were calculated. AAPC is a weighted average of the APCs which is a summary measure of the trend over a pre-specified fixed interval. If there were 0 joinpoint, the model would be a straight line and APC would equal to AAPC. When APC or AAPC is positive, it suggests an upward trend. Conversely, when APC or AAPC is negative, it offers a downward trend. When APC or AAPC is zero, it suggests a stable trend. The APC was tested using the Z test to determine if it was substantially different from zero. All hypothesis test was two-sided. *t*-test and Analysis of Variance (ANOVA) were used to compare the mortality between different age groups, genders, residences, and regionals with IBM spss25.0. The 0.05 level was used to determine statistical significance. Figures were drawn in origin 2022b.

To predict the ASMR until 2030, we assumed that the mortality trends from 2011 to 2020 would follow the linear variation obtained by the regression model for the next 10 years. We applied the estimates of the annual rate of change in mortality in the period 2011 to 2020 to the mortality rates from 2021 to 2030.

Python 3.10.4 was used to establish a linear regression model for the changes (percent) of annual mortality, which was calculated by the least square method. The projection formula was:

$$\beta ={\left(X^{T}X\right)}^{-1}X^{T}Y$$

$$\min_{\beta }{\left\|Y-X_{\beta }\right\|}^{2}$$

where β is regression coefficient, X is the time matrix, and Y is the standard mortality matrix. The difference equation used for prediction is as follows:

$$m_{t_{0}+t}=m_{t_{0}}\cdot \beta^{t}$$

where $m_{t_{0}+t}$ is the mortality rate that was predicted by our algorithm in the age t_0_+t, t is the time interval specified by the researcher.

### Patient and Public Involvement

Patient and public were not involved in the design, conduct, reporting, or dissemination of this research.

## Results

### CMR and the Trends of CMR, 2011–2020

**FIGURE 1:**
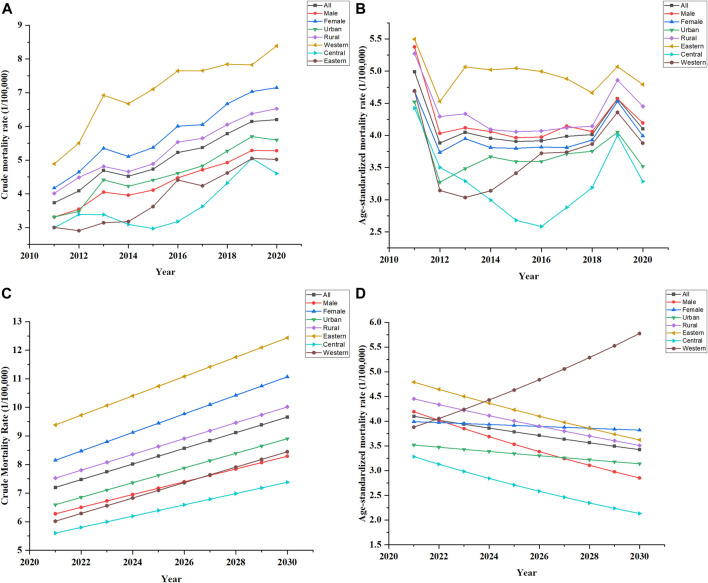
Trends in Crude mortality rate and Age-standardized rate, China. **(A)** Showed the past trends in crude mortality rate of all genders, residences, regions, 2011–2020. **(B)** Showed the past trends in age-standardized rate of all genders, residences, regions, 2011–2020. **(C)** Showed the future trends in crude mortality rate of all genders, residences, regions, 2021–2030. **(D)** Showed the future trends in age-standardized rate of all genders, residences, regions, 2021–2030.

CMR of AD and other dementias increased from 3.7 per 100,000 in 2011 to 6.2 per 100,000 in 2020, with an AAPC of 5.3% (95% CI 4.4%–6.3%) (Table 1). Females had higher CMR than males (*p* = 0.002). For both genders, CMR increased significantly with AAPC of 5.0% (95% CI 4.2%–5.9%) for males and 5.6% (95% CI 4.4%–6.7%) for females. There was a remarkable upward trend of CMR in both urban residents and rural residents with AAPC of 5.3% (95% CI 3.9%–6.7%) for urban and 5.4% (95% CI 4.4%–6.3%) for rural. Eastern residents had higher CMR than central and western residents (*p* < 0.001). CMR increased significantly for all regions with an AAPC of 7.3% (95% CI 5.5%–9.2%) for western, 6.6% (95% CI 3.2%–10.1%) for central 6.6% (95% CI 3.2%–10.1%) and 6.3% (95% CI 2.3%–10.5%) for eastern. CMR showed an upward trend in all genders, residences and regions (Figure 1A). In all regions, the CMR was always higher in rural areas than in urban areas. In particular, the CMR in 2020 was 8.98 per 100,000 in the eastern rural, while in the eastern urban was 7.4 per 100,000. The CMR in the western rural was 5.55 per 100,000 in 2020, and in the western urban was 4.16 per 100,000. Among all populations, females in the eastern rural had the highest CMR of 10.69 per 100,000 in 2020, while males in the western urban had the lowest CMR of 3.63 per 100,000.

**TABLE 1:** Crude Mortality Rate and the Joinpoint results in AD and other dementias by gender, residence, and region (China, 2011–2020).

	National			Urban			Rural			Eastern			Central			Western		
	All	Male	Female	All	Male	Female	All	Male	Female	All	Male	Female	All	Male	Female	All	Male	Female
2011	3.73	3.31	4.17	3.31	3.06	3.57	4.01	3.48	4.57	4.89	4.07	5.72	2.99	2.78	3.22	3.00	2.90	3.11
2012	4.09	3.55	4.65	3.48	3.00	3.96	4.49	3.90	5.11	5.51	4.51	6.53	3.39	3.14	3.65	2.91	2.66	3.17
2013	4.69	4.05	5.35	4.41	3.81	5.03	4.81	4.16	5.49	6.92	5.60	8.28	3.38	3.22	3.56	3.14	2.91	3.39
2014	4.52	3.96	5.11	4.23	3.68	4.78	4.66	4.09	5.26	6.67	5.57	7.81	3.09	2.84	3.35	3.18	3.04	3.32
2015	4.73	4.11	5.37	4.40	3.94	4.88	4.89	4.19	5.61	7.11	5.82	8.43	2.97	2.76	3.18	3.62	3.42	3.84
2016	5.22	4.47	6.01	4.61	4.02	5.21	5.53	4.69	6.41	7.65	6.26	9.07	3.18	2.90	3.46	4.41	3.95	4.89
2017	5.37	4.71	6.05	4.83	4.31	5.36	5.65	4.91	6.41	7.65	6.43	8.91	3.63	3.43	3.84	4.24	3.83	4.67
2018	5.78	4.93	6.67	5.27	4.50	6.06	6.05	5.15	6.99	7.85	6.35	9.39	4.31	3.90	4.74	4.62	4.15	5.11
2019	6.15	5.29	7.03	5.70	5.00	6.42	6.38	5.43	7.35	7.83	6.39	9.29	5.05	4.60	5.52	5.05	4.53	5.59
2020	6.20	5.28	7.15	5.60	4.67	6.55	6.53	5.61	7.48	8.39	6.83	9.99	4.60	4.02	5.20	5.02	4.62	5.44
APC (95% CI)	5.3* (4.4–6.3)	5.0* (4.2–5.9)	5.6* (4.4–6.7)	5.3* (3.9–6.7)	4.8* (3.3–6.3)	5.7* (4.1–7.3)	5.4* (4.4–6.3)	5.2* (4.3–6.0)	5.5* (4.4–6.6)	2011–2013 18.7 (−1.5–48.3)	2011–2013 18.2 (−6.5–49.4)	2011–2013 19.1 (−6.9–52.2)	6.6* (3.2–10.1)	5.8* (2.4– 9.4)	7.2* (3.8–10.8)	7.3* (5.5–9.2)	6.7* (5.3–8.2)	7.8* (5.5–10.2)
										2013–2020 3.0* (1.6–4.4)	2013–2020 2.9* (1.5–4.3)	2013–2020 3.0* (1.5–4.6)						
*p*-value	<0.001	<0.001	<0.001	<0.001	<0.001	<0.001	<0.001	<0.001	<0.001	0.106 0.002	0.126 0.003	0.128 0.003	0.002	0.004	0.001	<0.001	<0.001	<0.001
AAPC (95%CI)	5.3* (4.4–6.3)	5.0* (4.2–5.9)	5.6* (4.4–6.7)	5.3* (3.9–6.7)	4.8* (3.3–6.3)	5.7* (4.1–7.3)	5.4* (4.4–6.3)	5.2* (4.3–6.0)	5.5* (4.4–6.6)	6.3* (2.3–10.5)	6.1* (1.9–10.5)	6.4* (2.0–11.0)	6.6* (3.2–10.1)	5.8* (2.4–9.4)	7.2* (3.8–10.8)	7.3* (5.5–9.2)	6.7* (5.3–8.2)	7.8* (5.5–10.2)
*p*-value	<0.001	<0.001	<0.001	<0.001	<0.001	<0.001	<0.001	<0.001	<0.001	0.002	0.004	0.004	0.002	0.004	0.001	<0.001	<0.001	<0.001

*Significantly different from zero at the alpha = 0.05 level (*p* < 0.05).

AD, Alzheimer’s Disease; APC, annual percent change; AAPC, average annual percent change; CI, confidence interval.

CMR rose with age, peaking dramatically in the 85+ age group (Table 2), with dominant statistical significance of the whole age group (*p* < 0.001). CMR has ascending trends among the age group below 75 years with AAPC of 0.9% (95% CI −6.2%–8.5%) for 60–64, 0.5% (95% CI−2.0%–3.0%) for 65–69, and 2.2% (95% CI −2.9%–7.6%) for 70–74. And there were descending trends of CMR among the age group above 75 years with AAPC of −0.6% (95% CI −2.9%–1.8%) for 75–79, −0.7% (95% CI −2.4%–0.9%) for 80–84, and −0.1% (95% CI −4.4%–4.4%) for 85+. However, all the tendencies were not statistically significant. Below 85 years, males had higher CMR than females(*p* < 0.05), but above 85 years females had higher CMR than males (*p* = 0.33).

**TABLE 2:** Crude Mortality Rate and the Joinpoint results in AD and other dementias by age groups (China, 2011–2020).

	60–64			65–69			70–74			75–79			80–84			85+		
	All	Male	Female	All	Male	Female	All	Male	Female	All	Male	Female	All	Male	Female	All	Male	Female
2011	1.59	1.87	1.31	4.31	5.58	3.09	10.41	11.30	9.62	32.01	38.13	27.08	99.43	115.33	88.67	492.53	496.88	490.09
2012	1.90	2.23	1.56	4.63	5.46	3.81	11.57	13.66	9.64	31.20	32.70	29.92	89.67	90.50	89.04	304.01	302.19	305.12
2013	2.30	2.57	2.03	4.53	5.69	3.36	13.02	14.44	11.64	34.81	36.60	33.21	98.14	100.72	96.09	301.68	285.26	311.74
2014	2.39	2.81	1.96	5.31	6.29	4.33	12.12	13.93	10.35	30.21	31.66	28.91	83.73	89.01	79.49	284.86	262.02	299.05
2015	2.11	2.48	1.73	5.33	6.09	4.56	11.95	13.10	10.81	32.28	35.31	29.53	86.06	91.27	81.85	309.03	280.97	326.54
2016	2.36	2.75	1.96	4.94	5.75	4.13	11.80	13.34	10.31	29.21	31.55	27.13	83.61	84.61	82.82	324.61	303.79	337.58
2017	2.41	2.93	1.87	5.02	5.84	4.20	11.46	12.83	10.13	27.63	31.19	24.45	85.09	90.12	81.04	339.80	326.20	348.30
2018	2.43	2.87	1.98	5.07	5.61	4.54	12.50	13.89	11.15	26.29	28.25	24.53	83.57	86.73	81.01	347.95	326.09	361.66
2019	1.93	2.18	1.67	4.48	5.30	3.70	12.78	14.06	11.56	33.75	34.73	32.87	93.08	99.24	88.25	403.49	372.65	424.46
2020	1.76	1.84	1.68	4.50	4.74	4.26	12.32	13.76	10.95	33.12	36.99	29.72	91.91	97.25	87.78	336.92	315.61	350.56
APC (95% CI)	2011–2013 19.6 (−23.0–86.5)	2011–2013 17.6 (−27.3–90.1)	2011–2013 24.0 (−4.3–60.5)	2011–2015 5.5 (−0.1–11.4)	2011–2015 3.4 (−1.6–8.6)	2.5 (−0.5–5.5)	2011–2013 10.9 (−15.0–44.7)	0.9 (−0.8–2.7)	1.3 (−0.2–2.8)	−0.6 (−2.9–1.8)	−0.8 (−3.2–1.7)	−0.4 (−3.1–2.5)	−0.7 (−2.4–0.9)	−1.0 (−3.3–1.3)	−0.6 (−2.1–1.0)	−0.1 (−4.4–4.4)	2011–2013 −24.5* (−42.1–1.5)	0.3 (−3.8–4.6)
	2013–2018 0.9 (−12.4–16.1)	2013-2017 1.9 (−12.5–18.6)	2013–2020 −2.0 (−5.3–1.5)	2015–2020 −3.4 (−7.1–0.4)	2015–2020 4.5* (−7.7–1.1)		2013–2016 −3.2 (−25.8–26.3)										2013–2020 4.2* (0.6–8.0)	
	2018–2020 −14.8 (−45.4–32.9)	2017–2020 −20.4 (−50.8–28.6)					2016-2020 2.2 (−6.1-11.2)											
*p*-value	0.226	0.283	0.086	0.054	0.141	0.089	0.237	0.238	0.088	0.604	0.479	0.772	0.336	0.342	0.421	0.964	0.042	0.863
	0.819	0.649	0.196	0.071	0.019		0.652										0.030	
	0.261	0.177					0.384											
AAPC (95%CI)	0.9 (−6.2–8.5)	−0.4 (−8.0–7.7)	3.3 (−1.6–8.4)	0.5 (−2.0–3.0)	−1.1 (−3.2–1.2)	2.5 (−0.5–5.5)	2.2 (−2.9–7.6)	0.9 (−0.8–2.7)	1.3 (−0.2–2.8)	−0.6 (−2.9–1.8)	−0.8 (−3.2–1.7)	−0.4 (−3.1–2.5)	−0.7 (−2.4–0.9)	−1.0 (−3.3–1.3)	−0.6 (−2.1–1.0)	−0.1 (−4.4–4.4)	−3.0 (−7.7–2.0)	0.3 (−3.8–4.6)
*p*-value	0.812	0.914	0.192	0.716	0.344	0.089	0.407	0.238	0.088	0.604	0.479	0.772	0.336	0.342	0.421	0.964	0.234	0.863

*Significantly different from zero at the alpha = 0.05 level (*p* < 0.05).

AD, Alzheimer’s Disease; APC = annual percent change; AAPC, average annual percent change; CI, confidence interval.

### ASMR and the Trends of ASMR, 2011–2020

ASMR of AD and other dementias decreased slightly from 5.0 per 100,000 in 2011 to 4.1 per 100,000 in 2020, with AAPC of −0.4% (95% CI −2.5%–1.8%) (Table 3). Males had higher ASMR than Females (*p* = 0.019). ASMR reduced moderately for both sexes, with AAPC of −0.8% (95% CI −3.2%–1.6%) for males and −0.1% (95% CI −2.2%–2.1%) for females. Rural residents had higher ASMR than urban residents (*p* = 0.001). ASMR decreased in all residences, with AAPC of −0.5% (95% CI −2.8%–1.8%) for rural residents and −0.2% (95% CI −2.6%–2.2%) for urban residents. Eastern residents had higher ASMR than central and western residents (*p* < 0.001). The trend of AMSR for central residents with an AAPC of −1.6% (95% CI −6.5%–3.5%) decreased more rapidly than −0.2% (95% CI −4.0%–3.7%) of western residents and −0.6% (95% CI −1.9%–0.8%) of eastern residents. ASMR of urban females and Western females remained relatively stable. However, all the decreases of ASMR were not statistically significant.

**TABLE 3:** Age-standardized mortality rate and the Joinpoint results in AD and other dementias by gender, residence, and region (China, 2011–2020).

	National			Urban			Rural			Eastern			Central			Western		
	All	Male	Female	All	Male	Female	All	Male	Female	All	Male	Female	All	Male	Female	All	Male	Female
2011	4.99	5.38	4.69	4.52	4.80	4.29	5.27	5.77	4.91	5.50	5.80	5.25	4.42	5.03	4.02	4.69	5.08	4.35
2012	3.88	4.03	3.74	3.27	3.21	3.31	4.29	4.64	4.00	4.53	4.67	4.40	3.50	3.81	3.24	3.14	3.21	3.05
2013	4.05	4.12	3.95	3.48	3.49	3.44	4.34	4.44	4.21	5.07	5.02	5.05	3.29	3.56	3.03	3.03	3.18	2.90
2014	3.96	4.06	3.81	3.67	3.68	3.60	4.09	4.24	3.91	5.02	5.10	4.88	3.00	3.12	2.84	3.14	3.38	2.89
2015	3.91	3.97	3.80	3.59	3.72	3.45	4.06	4.09	3.97	5.05	4.97	5.03	2.68	2.85	2.51	3.41	3.65	3.18
2016	3.92	3.97	3.82	3.59	3.62	3.53	4.07	4.15	3.96	5.00	5.05	4.89	2.58	2.71	2.43	3.72	3.79	3.62
2017	3.99	4.15	3.81	3.71	3.84	3.58	4.12	4.30	3.92	4.88	5.04	4.70	2.88	3.14	2.64	3.74	3.87	3.61
2018	4.01	4.06	3.93	3.75	3.72	3.75	4.14	4.24	4.02	4.66	4.64	4.62	3.19	3.35	3.03	3.87	3.96	3.76
2019	4.57	4.57	4.53	4.05	4.08	4.01	4.86	4.86	4.82	5.07	4.92	5.14	4.00	4.15	3.86	4.36	4.49	4.23
2020	4.10	4.19	3.99	3.52	3.48	3.51	4.45	4.64	4.27	4.79	4.80	4.73	3.28	3.38	3.18	3.88	4.16	3.63
APC (95% CI)	−0.4 (−2.5–1.8)	−0.8 (−3.2–1.6)	−0.1 (−2.2–2.1)	−0.2 (−2.6–2.2)	−0.5 (−3.3–2.4)	0 (−2.1–2.1)	−0.5 (−2.8–1.8)	2011–2014 −10* (−17.4–1.9)	−0.1 (−2.4–2.2)	−0.6 (−1.9–0.8)	−1.1 (−2.5–0.4)	−0.3 (−1.7–1.2)	2011–2015 −11.6* (−21.0–1.1)	2011–2015 −12.8* (−22.4–2.0)	2011–2015 −11.2* (−20.8–0.3)	2011–2013 −17.7 (−29.0–4.6)	2011–2013 19.1* (−6.9-52.2)	2011–2013 −16.6 (−37–10.4)
								2014–2020 2.6 (−0.4–5.6)					2015–2020 7.2 (−1.0–16.1)	2015–2020 6.9 (−1.6–16.1)	2015–2020 7.8 (−0.6–17.0)	2013–2020 5.1* (3.0–7.2)	2013–2020 3.0* (1.5–4.6)	2013–2020 5.4* (1.5–9.4)
*p*-value	0.693	0.453	0.934	0.844	0.708	0.992	0.618	0.025 0.074	0.916	0.347	0.126	0.681	0.037 0.076	0.029 0.094	0.046 0.064	0.068 0.005	0.019 0.001	0.157 0.016
AAPC (95%CI)	−0.4 (−2.5–1.8)	−0.8 (−3.2–1.6)	−0.1 (−2.2–2.1)	−0.2 (−2.6–2.2)	−0.5 (−3.3–2.4)	0 (−2.1–2.1)	−0.5 (−2.8–1.8)	−1.8 (−4.4–0.8)	−0.1 (−2.4–2.2)	−0.6 (−1.9–0.8)	−1.1 (−2.5–0.4)	−0.3 (−1.7–1.2)	−1.6 (−6.5–3.5)	−2.4 (−7.4–2.9)	−1.1 (−6.1–4.2)	−0.2 (−4.0–3.7)	−0.5 (−3.2–2.3)	0 (−5.1–5.4)
*p*-value	0.693	0.453	0.934	0.844	0.708	0.992	0.618	0.178	0.916	0.347	0.126	0.681	0.53	0.369	0.684	0.916	0.74	0.995

*Significantly different from zero at the alpha = 0.05 level. (*p* < 0.05).

AD, Alzheimer’s Disease; APC, annual percent change; AAPC, average annual percent change; CI, confidence interval.

### Forecasted Trends in 2021–2030

If the trends continue, CMR in China would increase to 9.66 per 100,000 in 2030 (Figure 1C). CMR would increase to 8.91 per 100,000 in urban and 10.02 per 100,000 in rural. In males, CMR would increase to 8.29 per 100,000, while in females to 11.07 per 100,000. CMR would increase to 12.43 per 100,000 in the eastern population, 7.38 per 100,000 in the central population, and 8.45 per 100,000 in the western population.

ASMR in China would decline to 3.42 per 100,000 in 2030 (Figure 1D). ASMR would decline to 3.14 per 100,000 in urban and 3.51 per 100,000 in rural. ASMR would decline substantially in males to 2.85 per 100,000, while slightly in females to 3.82 per 100,000. A dramatic decline would occur in the central population to 2.13 per 100,000. A subtle decline would occur in the eastern population to 3.62 per 100,000. In contrast to other trends, the ASMR in the western population would rise to 5.78 per 100,000 in 2030.

## Discussion

Based on the national representative data, the study suggested that the mortality from AD and other dementias in China showed an upward trend from 2011 to 2020, which was in accord with domestic and foreign researches such as America, Japan, and South Korea [14, 18–20, 24]. The upward trend in CMR may be associated with the aging population, longer life expectancy, expanding medical insurance coverage areas, and changes in diagnostic criteria. The previous studies based on data of 2006–2012 suggested that ASMR among people aged 65 years and older decreased from 2006 to 2009 and increased up to 2012 [25]. Another study found that ASMR decreased in 2009–2015 [26]. In the study, the ASMR showed a downward trend in 2011–2020 [26]. After 2012, the ASMR was lower than the CMR, indicating the proportion of the elderly population was getting bigger. The decline in ASMR may be attributed to the improvement in education levels, the decrease in cerebrovascular diseases, and the complete prevention and treatment of related diseases. However, this did not mean that the burden of dementia will decrease, but rather suggested a further increase in population aging. This study also predicted that in 2021–2030 CMR would continue to increase, while ASMR would continue to decline except in the western area.

This study found that mortality of females was always higher than that of males, regardless of residence or region. According to The Global Heath Observatory (www.who.int), 65% of deaths from AD and other dementias are females. In previous epidemiological surveys, females had a higher dementia prevalence than males [27–29]. Moreover, females over 65 are almost twice as likely to develop AD as males of the same period [30], which is associated with longevity, genes, hormone levels, lifestyle, education, and social environment [31]. However, regardless of the age of onset, females have a longer median survival time [32]. Otherwise, females with other neurodegenerative diseases had a higher survival rate [33]. This may be connected with the protective effect of estrogen and the higher risk of comorbidities like cardiovascular and respiratory diseases in males. As this study showed that while females had higher CMR than males totally, males had a higher CMR than females before 85 years. And it may explain why females had higher CMR but lower ASMR than males.

Rural residents have higher mortality than urban residents. Domestic and international studies have pointed out urban-rural differences in dementia prevalence and mortality [34–37]. This difference is even more pronounced in China, where the majority of dementia was significantly higher in rural than in urban populations, and AD was the only subtype with a significant difference in prevalence between rural and urban [38]. China has a dualistic structure system of urban and rural areas, with apparent differences in income, medical resources, and health awareness, resulting in different effects on urban and rural healthcare. Previous studies have shown that education plays a crucial role in the development of dementia. People with higher education levels have a lower risk of developing dementia. The percentage of illiterate people over 65 in China was 48.2% in rural areas, compared with 17.7% in urban areas [38], which makes a more significant burden of dementia. The development of urbanization has led to a rising mobile population, with the proportion of the urban population rising by 14.21% and the nomadic population growing by 69.73% in the last decade. The flocking of young laborers to cities and the lack of elderly care services made the care of dementia patients a major problem. Although the access to health services has improved, under-diagnosis and under-management of dementia are still common. The mixed factors made the urban-rural difference even more apparent.

Consistent with previous studies [25, 26], we found that the eastern residents had higher mortality than central and western. With its excellent natural conditions and convenient transportation, the eastern area took the lead in development and became the most prosperous region in China with the support of state policies. Rapid economic growth has led to fast industrialization, urbanization, and aging, resulting in changes in ecological environments, disease profiles, disruption of work and rest patterns, and the development of irregular diets. These changes lead to an increased risk of developing chronic diseases. Hypertension, smoking, high sodium intake, and particulate matter pollution were the top four risk factors for both the number of deaths and the percentage of disability-adjusted life year loss in Chinese residents [39], and they were also associated with dementia. The prevalence of hypertension, diabetes, hyperlipidemia, overweight, and obesity was highest in East China. The combination of high-risk factors and comorbidities may explain why dementia mortality is noticeably higher in the east. This study found that from 2011 to 2020, the AAPC of the ASMR in western area was negative, showing a slight downward trend overall. And this trend will continue in the next decade. However, between 2013 and 2020, the APC was positive, showing a significant upward trend, and will continue in the next decade. This increase may due to the population growth, economic development and medical improvement brought by national policies such as “The Development of the Western Region” and “The Belt and Road.” In the next decade, more attention should be focused on the West China.

This study still has some limitations. Firstly, though CDC-DSP was nationwide and tightly regulated, it hardly covers the entire population of China. Secondly, due to the low diagnostic rate, the prevalence in this study may be underestimated, especially in rural areas. Thirdly, the National Death Surveillance System counts death cases according to the immediate cause of death. It is possible that people with AD or other dementias might die from other acute causes like cardiovascular disease or accidents. In that case, the system would code those conditions as the cause of death instead of AD or other dementias. And CDC-DSP cannot redistribute the garbage codes. Finally, it is worth mentioning that the elderly was afraid to go to hospital due to the impact of the COVID-19 pandemic, which would partially impact the data in 2019–2020. In addition, CDC-DSP has three monitoring sites in Wuhan which was the severely affected area. Both of the above could lead to an underestimation of the actual mortality rate.

In conclusion, this study showed the temporal trends in mortality for AD and other dementias in China from 2011 to 2020 and make a prediction for the next decade, suggesting a further development in the aging population and mortality from dementia in China. This study also reflected the age, gender, residence and regional differences, suggesting a change in future dementia work. The huge dementia population in China posed a great challenge to policymakers and healthcare professionals. Information provided by this study would help the relevant departments to provide better treatment and care services for patients by taking effective disease prevention and control measures.
